# No Evidence of Conpopulation Sperm Precedence between Allopatric Populations of House Mice

**DOI:** 10.1371/journal.pone.0107472

**Published:** 2014-10-08

**Authors:** Renée C. Firman, Leigh W. Simmons

**Affiliations:** Centre for Evolutionary Biology, University of Western Australia, Nedlands, Western Australia, Australia; University of Arkansas, United States of America

## Abstract

Investigations into the evolution of reproductive barriers have traditionally focused on closely related species, and the prevalence of conspecific sperm precedence. The effectiveness of conspecific sperm precedence at limiting gene exchange between species suggests that gametic isolation is an important component of reproductive isolation. However, there is a paucity of tests for evidence of sperm precedence during the earlier stages of divergence, for example among isolated populations. Here, we sourced individuals from two allopatric populations of house mice (*Mus domesticus*) and performed competitive *in vitro* fertilisation assays to test for conpopulation sperm precedence specifically at the gametic level. We found that ova population origin did not influence the outcome of the sperm competitions, and thus provide no evidence of conpopulation or heteropopulation sperm precedence. Instead, we found that males from a population that had evolved under a high level of postcopulatory sexual selection consistently outcompeted males from a population that had evolved under a relatively lower level of postcopulatory sexual selection. We standardised the number of motile sperm of each competitor across the replicate assays. Our data therefore show that competitive fertilizing success was directly attributable to differences in sperm fertilizing competence.

## Introduction

In many species females solicit copulations from multiple partners to generate competition among the ejaculates of rival males [1], [2]. In the absence of strict monogamy, the reproductive interests of males and females will not align, and thus lead to antagonistic coevolution between the sexes [3]. Sexual conflict has been shown to lead to the rapid evolution of antagonistic characters [4], [5], and arises at the gametic level when the sperm of different males compete for fertilizations [6]. Sexual conflict theory suggests that after periods of allopatry, heteropopulation males might be more successful during sperm competition because females will have evolved ‘resistance’ to conpopulation males [3], [7]. This outcome will impede speciation as gene flow is enhanced.

Alternatively, males from the same population as females will have a competitive advantage over heteropopulation males when they are *better* adapted to females and their defenses. Under this scenario sperm precedence will reduce gene flow, and create reproductive barriers that may eventually lead to the evolution of new species [8]. While postcopulatory barriers to gene exchange can occur either before (prezygotic) or after (postzygotic) the fusion of the sperm and egg, current research is biased toward postzygotic barriers because prezygotic barriers are physiological and subtle, and therefore difficult to investigate. Consequently, little is known about the isolating mechanisms between insemination and fertilization.

The phenomenon of sperm precedence has been best studied at the interspecific level. In the absence of precopulatory discrimination, polyandrous females in hybrid zones will mate with both conspecific and heterospecific males. When the ejaculates of males of different species co-occur in the female tract, a fertilization bias toward male/s of the same species as the female can occur via the phenomenon of conspecific sperm precedence (reviewed in [9]). For internal fertilizers, conspecifics will experience a competitive advantage when heterospecific sperm fail to reach the site of fertilization (reduced sperm transit) or fail to fertilize the egg (gametic incompatibility). For example, the female tract might eliminate the effective transfer of heterospecific sperm to storage organs [10]–[13], or heterospecific sperm might fail to leave storage organs due to their inactivation by the seminal fluid of rival conspecific sperm [14], [15]. Moreover, reproductive proteins diverge rapidly [16]–[19] and males and females coevolve quickly for fertilization events [6]. Consequently, in the situation when both heterospecific and conspecific sperm occur simultaneously within the vicinity of the ova, preferential fertilization by conspecific sperm would occur when heterospecific sperm-ova interactions are deemed incompatible due to non-connectivity in gamete signalling and/or sperm attachment [9].

Conspecific sperm precedence has been observed in a number of different species (e.g. [10], [13], [20]–[24]). However, conpopulation sperm precedence, where males from the same population as the female achieve higher fertilization rates than expected by chance compared to males from different populations, is less well evidenced [9]. Studies of conpopulation sperm precedence using insects have produced varying results, and biases in favour of the production of both conpopulation and heteropopulation offspring have been observed [12], [25]–[27]. Evidence among vertebrate taxa is currently limited to only a single study in which conpopulation males were observed to have a competitive fertilization advantage over heteropopulation males (*Poecilia reticulata*; [28]). Such data is critical for providing insight into how quickly postcopulatory, prezygotic reproductive barriers might evolve.

In this investigation we assessed whether conpopulation sperm precedence was evident in two allopatric populations of the house mouse, *Mus domesticus*. First, we conducted monogamous crosses between mice from different populations to establish compatibility between populations *in vivo*. Second, we performed competitive *in vitro* fertilization (IVF) assays between the sperm of males from (i) the same population as the ova donors, and (ii) a different population to the ova donors. In so doing, we were able to test directly for the presence of gametic postcopulatory, prezygotic reproductive barriers that might prevent fertilizations by heteropopulation sperm. Alternatively, a fertilization bias toward heteropopulation males could arise if females were less ‘resistant’ to males with which they have coevolved, and thus provide evidence of sexually antagonistic coevolution [26], or female ‘selection’ of genetically dissimilar sperm to obtain fitness benefits by producing high quality, hybrid offspring [29], [30].

## Materials and Methods

### Ethics statement

This study was carried out in strict accordance with the Australian Code of Practice for the Care and Use of Animals for Scientific Purposes. The protocol, including the field work, was approved by the University of Western Australia Animal Ethics Committee (approval number: 07/100/607). The house mice used in these experiments were sacrificed via an intra-peritoneal injection (>160 mg/kg) of pentobarbitone.

### Experimental animals

Male (20) and female (20) house mice (*Mus domesticus*) were trapped in Elliot small mammal traps (baited with peanut butter and rolled oats) on Whitlock Island (30°19'S, 114°59'E) and Rat Island (28°42'S, 113°47'E) located off the coast of Western Australia. Rat Island is part of the Abrolhos Archipelago, and Whitlock Island is within Jurien Bay. The exact source of these mouse populations is currently unknown, but presumed to have been established by stowaways on European ships that wrecked on the islands during the 1600 s [31]. The mice were housed individually in boxes during transfer from the field sites to the University of Western Australia. In the laboratory the animals were maintained in a constant temperature room (24°C) on a reversed 14∶10 hour light-dark cycle. Food and water was provided *ad libitum*. The animals were outbred under a common-garden breeding regime to eliminate any environmental factors that might induce plasticity in phenotype. Male and female pairs were housed in a box for a maximum of two weeks, or until the female was noted as being pregnant. Litters were weaned at three weeks of age, at which time males were housed individually and females were housed in sibling groups. Each population colony was bred under these common-garden conditions for five generations before being used in this experiment.

These populations had previously been shown to differ in the expected frequency of multiple paternity, corresponding to high (71% of litters multiply sired; Rat Island) and low (17% of litters multiply sired; Whitlock Island) levels of postcopulatory sexual selection [32].

### Interpopulation reproductive success

Monogamous matings between individuals of the Whitlock Island and Rat Island populations were established to test for precopulatory isolation and reproductive failure. Thus, 10 Rat Island population males were paired with 10 Whitlock Island population females (R-♂ × W-♀), and 10 Whitlock Island population males were paired with 10 Rat Island population females (W-♂ × R-♀). Females were checked daily for the presence of a mating plug, which is indicative of a successful mating [33]. When a plug was observed, females were separated from the male and supplied with shredded paper for nesting. Approximately 17 days after mating females were sacrificed, dissected, and scored for pregnancy, and embryo number was recorded.

### Competitive IVF assays

The competitive IVF assays were performed as described previously [24]. Ova were retrieved from the oviduct ampullae of females that had been induced to superovulate via a series of hormone injections [24]. Epididymides were removed from males, placed in 1 mL drops of Human Tubal Fluid (HTF), and incubated at 37°C under 5% CO_2_/air for 10 minutes to allow sperm to disperse into the medium [24]. The sperm suspensions were stained with DNA flurochromes Hoechst 33342 (Invitrogen) or SYTO 82 orange (Invitrogen). Final concentrations used for sperm labelling were 4.05µM Hoechst 33342 and 15.00µM STYO 82 orange. Sperm were labelled during a 20 minute incubation at 37°C. Excess dye was removed by centrifugation at 3000 rpm ( =  300 ×*g*) for eight minutes and resuspended in HTF. Sperm were then capacitated at 37°C under 5% CO_2_/air [34].

The IVF assays were performed in 500 µL drops of HTF under mineral oil. The ova from three females, drawn from the same population, were pooled in a single assay. In each assay, the ova were mixed with the sperm from both a high and low sperm competition population male. We controlled for differences in sperm number and percentage motility between populations by mixing the same number of motile sperm from each male in each competitive assay. The concentration of motile sperm in each dyed, resuspended suspension was determined using a computer assisted sperm analyser (CASA), following which an aliquot was removed and added to the IVF assay to give a final concentration of 1.0 × 10^6^ motile sperm/mL. We alternated using the Hoechst 33342 and STYO 82 orange to stain the sperm of males from the two different populations across the replicate assays. The gametes were coincubated for 4 hours at 37°C under 5% CO_2_/air, following which the ova were washed in 100 µL drops of HTF to remove cumulus cells or attached sperm. The ova were then viewed under a Zeiss Axio Imager.A1 fluorescent microscope (400 ×) and scored as being fertilized by either a Hoechst 33342 labelled sperm (380 nm filter) or a STYO 82 orange labelled sperm (560 nm filter).

## Results

### Interpopulation reproductive success

The interpopulation monogamous male-female pairs initiated reproduction normally. Of the 20 pairs, 80% mated within the first seven days of being paired and all had mated within 12 days, which is comparable to latencies observed among intrapopulation crosses performed under identical conditions [unpub. data]. However, the pregnancy rate differed among females from the two populations, with a smaller proportion of the R-♂ × W-♀ matings (30%) resulting in pregnancies, compared to the W-♂ × R-♀ matings (80%) (χ^2^  =  5.05, df  =  1, *P*  =  0.03). Embryo numbers were comparable among females of the two populations (*F*_1, 9_  =  0.12, *P*  =  0.74; mean: W  =  6.0 ± 0.6, R  =  6.4 ± 0.6), and equivalent to those reported for intrapopulation crosses (in the wild, [32]; in the laboratory, [unpub. data]).

### Competitive IVF assays

We used a generalised linear mixed model (GLMM) fit by the Laplace approximation using the ‘lme4’ library in the *R*-statistical analysis package to test for conspecific sperm precedence [35]. Thus, the number of ova fertilized by the Rat Island male was assigned to be the response variable, total number of fertilized ova was assigned as the binomial *n*, and assay ID was modelled as a random effect. The GLMM revealed that ova population origin did not influence the fertilization success of the different sperm types among the replicate IVF assays, thus providing no evidence of conpopulation sperm precedence (Table 1).

**Table 1:** No evidence of conspecific sperm precedence.

Random effects	Variance	Std. Dev.
Assay ID	0.434	0.659

Fixed effects	Estimate	s.e.	*z*-value	*P*
(Intercept)	1.059	0.309	3.429	<0.001
Ova population origin	0.294	0.442	0.665	0.506

A GLMM fit by the Laplace approximation (*R*) revealed that the population from which the ova where derived had no effect on the proportion of ova fertilized by the sperm of males from the Rat Island population.

We assessed whether competitive fertilization rates differed among the sperm of males from the different populations. As there was no effect of ova origin, we pooled the replicate assays and applied a single factor, matched pairs *t*-test to compare the proportion of ova fertilized by the Rat Island male against an expectation of equal fertilization success (0.5). The analysis revealed that males from the Rat Island population achieved more fertilizations than expected by chance (*t*_15_  =  6.346, *P* <0.001, mean  =  0.74 ± 0.04; Table 2).

**Table 2:** Data from 16 competitive IVF assays.

	(a) Total ova number		(b) Number (%) ova fertilized		(c) Success of Rat Island male	
Ova donors' population	Rat (High)	Whitlock (Low)	Rat (High)	Whitlock (Low)	Rat (High)	Whitlock (Low)
	21	19	17 (80)	15 (79)	0.82	0.80
	14	13	11 (79)	13 (100)	0.82	0.85
	19	23	14 (74)	21 (91)	0.64	0.86
	12	28	10 (83)	23 (82)	0.50	0.52
	22	47	18 (82)	44 (94)	0.78	0.98
	24	16	22 (92)	14 (88)	0.73	0.57
	17	9	17 (100)	9 (100)	0.65	0.56
	35	18	33 (94)	18 (100)	0.88	0.89
**Mean ± s.e. (total)**	20.5 ± 2.5 (164)	21.6 ± 4.2 (173)	17.8 ± 2.6 (142)	19.6 ± 3.8 (157)	0.72 ± 0.04	0.75 ± 0.06

Two replicate assays where run in parallel, one for which ova were donated by females from the Rat Island (high-level postcopulatory sexual selection) population and the other from the Whitlock Island (low-level postcopulatory sexual selection) population. Across the replicate assays, the total number of ova ovulated (ANOVA: *F*_1, 14_  =  0.054, *P*  =  0.821; a) and the proportion of ova that were fertilized (GLMM: *F*_1, 14_  =  0.837, *P*  =  0.376; b) did not differ between ova from females of the different populations. The success of males from the Rat Island (high-level) population is expressed as proportions of the total number of fertilized ova (c).

## Discussion

When males and females of different species mate, fertilization success may be low due to poor storage or transport of sperm [10]–[13], or because of incompatible gamete interactions [36]–[38]. Gametic incompatibility has been observed to operate as an effective isolating mechanism among different taxa, and may be driven by a coevolutionary arms race between sperm and ova [24]. However, interspecific, cross-fertilization experiments have provided evidence that reproductive ‘isolation’ at the gametic level is often incomplete, and can be asymmetric [24], [39], which begs the question how rapidly does gametic isolation arise?

An investigation that utilised IVF provided evidence of conspecific sperm precedence among three different *Mus* species [24]. It was shown that conspecificity was lower for species pairs where the male was of a species that had evolved under high levels of postcopulatory sexual selection, suggesting that sperm competition selects for ‘aggressive’ sperm and their ability to overcome the ovum barriers of closely related species [24]. Two of these species (*M. spretus* and *M. domesticus*) exist in sympatry, however precopulatory isolating mechanisms preclude them from producing hybrids in nature [40], [41]. In contrast, two other closely related species, *M. musculus* and *M. domesticus*, hybridize in a narrow zone where their European distributions overlap [42]. A series of competitive IVF assays revealed that the sperm of *M. musculus* consistently outcompeted the sperm of *M. domesticus* regardless of which species donated the ova [43]. The results of our intraspecific investigation parallel these findings; we found no evidence of conpopulation sperm precedence between two allopatric populations of *M. domesticus*. Nor did we find evidence of a heteropopulation sperm advantage. Instead, the sperm of males from the Rat Island population consistently had a competitive advantage over the sperm of Whitlock Island males regardless of the population origin of the ova. Thus, whilst gametic isolation appears to exist among distantly related species of *Mus* [44], and the magnitude of sperm conspecificity correlates with species' levels of postcopulatory sexual selection [24], current evidence suggests that postcopulatory reproductive barriers do not occur between closely related *Mus* species [43], or among populations at the intraspecific level.

The competitive advantage to Rat Island males could be attributable to postcopulatory sexual selection and the evolution of sperm fertilizing competency. High sperm numbers and high sperm motility are important determinants of competitive fertilization success in vertebrates [45]–[50]. Males from the Rat Island population, which have evolved under a high level of postcopulatory sexual selection, are known to produce ejaculates with greater numbers of sperm and higher proportions of motile sperm compared to males from the Whitlock Island population, which have evolved under a low level of postcopulatory sexual selection [32], [51]. Here, we controlled for these differences by inseminating each replicate IVF assay with an equivalent concentration of motile sperm, allowing us to directly assess the relationship between the relative strength of postcopulatory sexual selection and other aspects of sperm fertilizing competence. For example, mammalian sperm need to undergo the process of “capacitation” to be competent to fertilize an ovum, and a positive correlation between the strength of postcopulatory sexual selection and the proportion of sperm that undergo capacitation has been documented among *Mus* species [52]. Therefore, differences in the regulation of the sperm capacitation process provides one explanation for the pattern of competitive fertilization success that we observed among males from these two populations.

Alternatively, the strength of postcopulatory sexual selection has also been reported to influence sperm responsiveness to ovum signals, and consequently the speed at which they fertilize [52]. This scenario has important implications regarding the potential for an increased risk of polyspermy, and adjustments in ovum resistance to fertilization [6], [53]. Importantly, our results suggest that postcopulatory sexual selection influences more than just the evolution of sperm traits that contribute to reaching the site of fertilization, but also traits that contribute to their functional capacity once they arrive [52].

In our *in vivo* trials we found considerable reproductive failure when Rat Island males mated with Whitlock Island females. Failure of implantation due to impaired embryo quality could account for the low pregnancy rate. The asymmetry in reproductive failure rate could be the result of incompatibilities between maternal and paternal genotypes. For example, sex chromosomes, imprinted genes, and mtDNA are all potential candidates for genetic incompatibilities arising from interactions between maternal and paternal genomes during post-fertilization events, including interpopulation ‘hybrid’ embryo development. Alternatively, the asymmetry in reproductive compatibility could be attributable to polyspermy, which results in embryo mortality [53]. Previously, we had found that females from the Whitlock Island population produce ova that are more easily fertilized than ova produced by females from the Rat Island population [54]. Consequently, the coupling of ‘aggressive’ Rat Island male sperm with low ‘resistant’ Whitlock Island female ova could have generated an unusually high rate of polyspermic fertilizations and embryo death in these crosses.

In conclusion, we performed sperm competitions under controlled *in vitro* conditions, and assessed the competitive ability of sperm and the selective ability of ova in the absence of extrinsic factors, such as those that influence sperm transit to the site of fertilization. Despite there being some degree of reproductive isolation at the interspecific level among *Mus* species [24], we report no evidence of conpopulation sperm precedence between two populations of the house mouse, *M. domesticus*. Our adopted methodologies allowed us to conduct a rigorous assessment of gametic isolation, however other mechanisms of postcopulatory, prezygotic barriers might also exist between these populations. Indeed, *in vivo* fertilization has the potential to be mediated by interactions between sperm, the seminal fluid, and the female reproductive tract [43]. Our *in vivo* crosses suggest asymmetric reproductive barriers, which may be due to gametic interactions and/or other interactions between sperm and the female reproductive tract. Finally, we found that males from a population subject to relatively high levels of postcopulatory sexual selection had significantly greater competitive fertilization success compared to males from a population subject to relatively low levels of postcopulatory sexual selection, irrespective of the population from which ova were sourced. Our data thus adds to growing evidence that selection via sperm competition improves sperm fertilizing competency to maximise male fertility.
